# Why Does It Hurt So Much? Emotion Regulation Mediates the Association between Fibromyalgia Symptoms and Psychological Distress

**DOI:** 10.3390/healthcare11101368

**Published:** 2023-05-10

**Authors:** Lee Frumer, Hadar Marom Harel, Danny Horesh

**Affiliations:** 1 Department of Psychology, Bar-Ilan University, Ramat-Gan 5290002, Israel; 2 Maccabi Healthcare Services, Tel Aviv 68125, Israel; 3 Department of Psychiatry, New York University Grossman School of Medicine, New York, NY 10016, USA

**Keywords:** fibromyalgia, stress, depression, emotion regulation, comorbidity

## Abstract

While it is known that fibromyalgia patients often suffer from depression and stress symptoms, there is inconclusive evidence as to why these symptoms occur. The aim of this study is to examine the role of emotion regulation in mental health symptoms among treatment-seeking individuals with fibromyalgia. Ninety-three (93) participants (mean age = 47.25, SD = 12.4) were recruited from one of Israel’s largest community healthcare providers. They were administered self-report questionnaires assessing fibromyalgia (FIQR), perceived stress (PSS), major depression (PHQ-9), and difficulties in emotion regulation (DERS). Associations were found between measures of fibromyalgia symptoms, psychological distress, and emotion regulation. Several sub-indices of emotion regulation showed significant correlations with psychological distress, with non-acceptance of emotional responses showing the strongest associations. Moreover, non-acceptance of emotion responses mediated the association between fibromyalgia symptoms and psychological distress. Our findings show that the connection between fibromyalgia symptoms and psychological distress is partially explained by difficulties in emotion regulation. Moreover, we show that specific emotion regulation strategies play a differential role in fibromyalgia patients’ distress, thereby highlighting the importance of identifying unique psychotherapeutic targets. Specifically, regulating emotions through acceptance of emotional responses seems to be particularly important for fibromyalgia patients, as they cope with stigma and a lack of validation.

## 1. Introduction

Fibromyalgia is a clinical disorder predominant in females with an unknown etiology and medically unexplained symptoms (MUS) [1,2]. It is characterized by widespread skeletal muscle pain with no obvious tissue pathology [1,3]. People with fibromyalgia often experience high levels of fatigue, memory deficits, and mood difficulties [1,3]. Fibromyalgia patients often suffer from psychiatric comorbidity [2,4,5] and express feelings of embarrassment, frustration, guilt, isolation, and shame [6].

Fibromyalgia is often classified as a functional disorder, a term that refers to a group of disorders previously designated as psychosomatic or somatoform in nature, owing to the absence of a clear physical pathology. Most models of the disorder propose a biopsychosocial etiology, in which physiological, psychological, and social factors contribute to illness onset [7]. As such, fibromyalgia has been conceptualized as a ‘centralized pain state’ that can be triggered and maintained by stressful life events [1]. There are studies that support the idea that people with fibromyalgia endure increased levels of distress in daily life and have been more frequently exposed to stressful and traumatic life events compared to healthy individuals [7,8].

Emotion regulation has been conceptualized in various ways. One view emphasizes the control of emotional expression and the reduction of emotional arousal [9]. Gross, for example, addresses different emotional regulation mechanisms and emphasizes the positive value of re-appraisal versus the less adaptive role of suppression [10]. In recent years, with the advent of “third wave” cognitive-behavioral approaches, researchers have also emphasized the importance of awareness and acceptance of negative feelings/cognitions in promoting emotion regulation [11].

Despite high rates of emotional distress among fibromyalgia patients, few studies have addressed processes of emotion regulation in this population. Various studies show that awareness (attention, differentiation, and labeling of emotion), expression (avoidance or suppression vs. expression of emotion), and experiencing (accessing, experiencing, and reflecting on one’s emotions to enhance adaptation) of negative emotions are associated with, and likely contribute to, greater physical pain, symptoms, and dysfunction among patients diagnosed with medically unexplained symptoms (MUS) [12,13]. Studies have reported low levels of positive emotions [14] and high levels of negative emotions [15], as well as a sense of being emotionally overwhelmed among those with fibromyalgia [16]. In addition, high affect instability was associated with increased pain-related disability [15]. As for emotion regulation strategies, fibromyalgia patients have been found to experience difficulties recognizing, accepting, expressing, and clarifying emotions while also over-utilizing emotional avoidance [17,18,19]. A novel model suggested that an imbalance in emotion regulation, reflected by an overactive “threat” system and an underactive “soothing” system, might keep the “salience network” (also known as the midcingulo-insular network) in continuous alert mode, and this hyperactivation, in conjunction with other mechanisms, contributes to fibromyalgia [8]. In addition, following the model proposed in the article, the researchers suggest that fibromyalgia patients can benefit from interventions that reinforce soothing abilities, which include acceptance, compassion, and more [8].

While it is known that fibromyalgia patients often suffer from depression, anxiety, and stress [1,2,3], there is inconclusive evidence as to why these symptoms occur [7]. To the best of our knowledge, no study to date has explored the role of emotion regulation as a mediator in the relationship between fibromyalgia symptoms and psychological distress [6,16]. In addition, there is a scarcity of research around the specific components of emotion regulation that may explain psychological distress in fibromyalgia [16]. The present study aims to bridge these gaps in research. The aim of this study is to examine the role of emotion regulation difficulties in mental health symptoms among treatment-seeking fibromyalgia patients. We hypothesized that the level of depression and perceived stress symptoms would be positively associated with fibromyalgia symptoms, while Health-Related Quality of Life (HRQOL) would be negatively associated with fibromyalgia symptoms. We also hypothesized that the above associations would be mediated by difficulties in emotion regulation, i.e., higher fibromyalgia symptom severity would be associated with more difficulties in emotion regulation, which, in turn, would be associated with increased psychological distress/decreased HRQOL.

## 2. Materials and Methods

### 2.1. Participants and Procedure

Participants in this study were patients diagnosed with fibromyalgia who applied to “Maccabi Healthcare Services” (MHS, a large nationwide healthcare provider in Israel) mental health clinic to attend a psychotherapy clinical trial. Ethical approval had been obtained from the MHS ethics committee (ASMC-0111-18 04/04/2019), and all participants signed informed consent forms. Participants were included if they were 18 years of age or older, had a formal fibromyalgia diagnosis given by an expert rheumatologist, and were Hebrew speakers. Exclusion criteria included active suicidality, psychosis, substance abuse, or a significant cognitive limitation.

Ninety-two (92) participants met the inclusion criteria. A total of 86 (93.5%) were women, and the mean age was 47.25 (SD = 12.4). A total of 92 (100%) were Jewish. An amount of 41 (44.5%) reported being in a relationship, and 63 (68.4%) had an academic education. Only 28 (30.4%) reported working full time. A total of 22 (23.9%) reported income below average, and 36 (39.1%) reported income above average.

### 2.2. Measures

The sociodemographic questionnaire included questions about participants’ age, gender, level of education, family status, religion, and income.

The Fibromyalgia Impact Questionnaire Revised (FIQR) [20] is a self-report questionnaire that includes 21 items. It assesses symptoms of fibromyalgia with reference to functioning, overall impact, and symptom severity. Questions are rated on a 0 (no difficulty) to 10 (very difficult) scale. The scale was found to have good psychometric properties [20]. In the current study, the FIQR demonstrated excellent internal consistency, with a Cronbach’s alpha of 0.93.

The Perceived Stress Scale (PSS) [21] is a self-reported 10-item questionnaire assessing the extent to which events in the past month were perceived as unexpected, uncontrollable, and overwhelming. Items are rated on a scale ranging from 0 (never) to 4 (very often). Scores can also be divided according to norms: low-moderate perceived stress: 0–26, and high perceived stress: 27–40 [22]. Previous studies showed the PSS to have excellent psychometric properties [21]. In the current study, the PSS demonstrated very good internal consistency, with a Cronbach’s alpha of 0.88.

The Patient Health Questionnaire-9 (PHQ-9) [23] is a self-report questionnaire assessing major depression symptoms by rating each of the 9 DSM-IV symptoms from 0 (not at all) to 3 (nearly every day). It can yield a continuous score and a probable major depressive disorder diagnosis using a cut-off of 10 [23]. Previous studies have found the PHQ-9 to have excellent psychometric properties [23]. In the current study, the PHQ-9 demonstrated very good internal consistency, with a Cronbach’s alpha of 0.84.

The Difficulties in Emotion Regulation Scale (DERS) [24] is a self-reported questionnaire assessing emotion regulation difficulties. It comprises 36 items assessing six domains: Non-acceptance of negative emotions, inability to engage in goal-directed behaviors, difficulties controlling impulsive behaviors, limited access to effective emotion regulation strategies, lack of emotional awareness, and lack of emotional clarity. Responses are given on a 5-point Likert scale from 1 (almost never) to 5 (almost always). The questionnaire was found to have good psychometric properties [24]. In the current study, DERS demonstrated very good internal consistency, with a Cronbach’s alpha of 0.87.

### 2.3. Data Analysis

In order to calculate the associations between study variables, we have calculated Pearson correlations. In order to examine emotion regulation by major depression and perceived stress levels, we conducted a Multivariate Analysis of Variance (MANOVA). For the mediation analysis, Model 4 of the PROCESS macro for SPSS [25] was employed twice—once for each outcome measure (MDD and perceived stress).

## 3. Results

### 3.1. Associations between Study Variables

Table 1 presents Pearson correlations calculated to assess the association between emotion regulation and the severity of physical and psychological symptoms. As can be seen in the table, these significant positive correlations were found between all measures of fibromyalgia and psychological distress. In addition, positive and significant correlations were found between these measures and all emotion regulation subscales, except for lack of emotional awareness and lack of emotion clarity. The strongest correlations were found between non-acceptance of emotional responses and all three outcome measures (MDD, stress, and fibromyalgia; see Table 1).

### 3.2. ER According to Major Depression and Perceived Stress Levels

Since the PHQ-9 has a cutoff score for a probable MDD diagnosis ≥ 10 [23]. We also examined the differences in emotion regulation between those above/below the threshold. 73 participants (78.5%) met the probable MDD cutoff. Multivariate Analysis of Variance indicated a difference in emotion regulation score between those below (M = 91.41, SD = 25.24) and above (M = 109.63, SD = 21.87) the cutoff (F_1,93_ = 9.02, *p* < 0.01). One-way ANOVA testing indicated significant differences in the following emotion regulation subscales: Nonacceptance of emotional responses (below cutoff: M = 12, SD = 4.92; above: M = 18.66, SD = 6.56; F_1,93_ = 15.46, *p* < 0.001), difficulty engaging in goal-directed behavior (below: M = 13.88, SD = 3.33; above: M = 16.82, SD = 4.13; F_1,93_ = 7.41, *p* < 0.01), impulse control difficulties (below: M = 13.05, SD = 2.16; above: M = 17.13, SD = 4.96; F_1,93_ = 10.90, *p* < 0.001), and limited access to emotion regulation strategies (below: M = 18.52, SD = 5.25; above: M = 23.50, SD = 6.92; F_1,93_ = 7.70, *p* < 0.01). A Bonferroni–Holm [26] correction procedure was applied to adjust for multiple comparisons.

The PSS similarly has validated cutoff scores [22]. Thus, we found a difference in emotion regulation scores between participants with low-moderate (PSS ≤ 26) (M = 99.27, SD = 19.31) and high perceived stress (PSS ≥ 27; M = 120.68, SD = 24.21). One-way ANOVA testing indicated significant differences in the following subscales: Nonacceptance of emotional responses (mild; M = 14.82 SD = 5.70) moderate-severe (M = 22.56 SD = 5.64) (F_1,93_ = 37.57, *p* < 0.001), difficulty engaging in goal-directed behavior (mild (M = 15.61 SD = 3.84), moderate-severe (M = 18.14, SD = 4.39) (F_1,93_ = 7.91, *p* < 0.01), impulse control difficulties (mild; M = 14.91 SD = 3.86) moderate-severe (M = 19.23 SD = 5.33) (F_1,93_ = 19.66, *p* < 0.001), limited access to emotion regulation strategies (mild; M = 20.62 SD = 5.63) moderate-severe (M = 26.59 SD = 7.41)) (F_1,93_ = 18.52, *p* < 0.001), and lack of emotional clarity (mild; M = 12.69 SD = 2.28) moderate-severe (M = 14.50 SD = 3.87) (F_1,93_ = 7.98, *p* < 0.01). Here too, all results were statistically corrected for multiple comparisons using the Bonferroni–Holm correction [16].

### 3.3. The Mediating Role of Emotion Regulation

First, fibromyalgia total score was the independent variable, major depression was the dependent variable, and difficulties in emotion regulation were the mediators. For the second test, fibromyalgia was the independent variable, perceived stress was the dependent variable, and difficulties in emotion regulation were the mediators. The emotional regulation variable was entered in two separate models, first in the sum and then in the different sub-indices.

The predictive model for PSS (R^2^ = 0.74, F [1,91] = 55.15, *p* < 0.001) showed a significant effect for DERS total score (β = 0.08, *p* < 0.001) and for FIQR total score (β = 0.20, *p* < 0.001). Estimates of the indirect effect for DERS total score (β = 0.03) had a CI (0.01–0.08) that was significantly greater than zero, indicating that DERS total score partially mediated the path between FIQR total score and PSS total score. The predictive model for MDD was not statistically significant.

**Figure 1:**
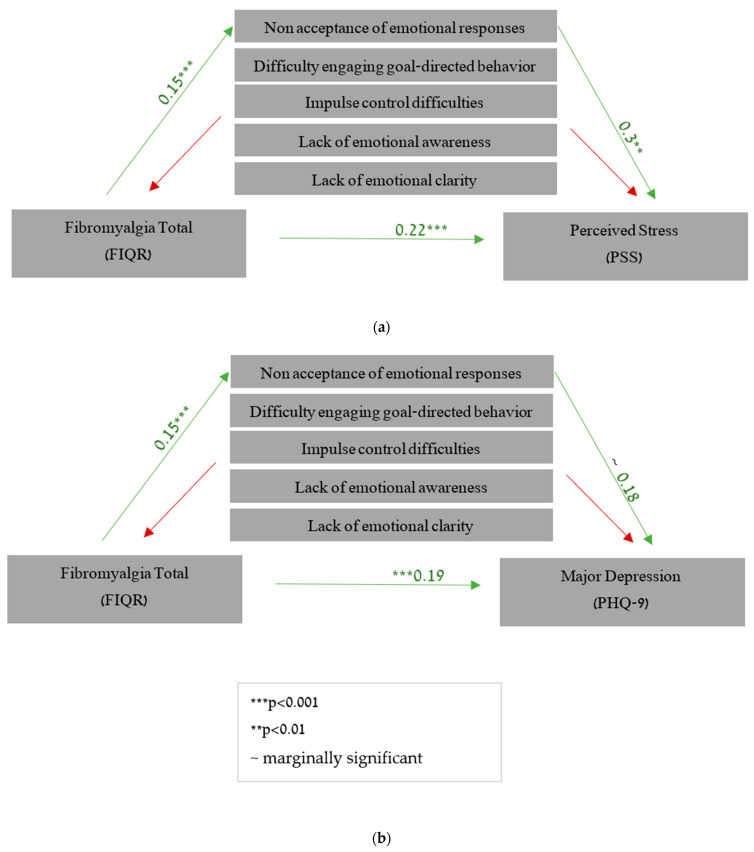
(**a**) The mediating role of emotion regulation in the association between FM symptoms and perceived stress. (**b**) The mediating role of emotion regulation in the association between FM symptoms and major depression.

Next, we tested the specific DERS subscales as mediators. The predictive model for PSS as the outcome. The predictive model (R^2^ = 0.81, F [7,85] = 23.45, *p* < 0.001) showed a significant effect for the non-acceptance score (β = 0.30, *p* < 0.01) and for the FIQR total score (β = 0.22, *p* < 0.001). Estimates of the indirect effect of the non-acceptance score (β = 0.04) had a CI (0.01–0.09) that was significantly greater than zero, indicating that non-acceptance score partially mediated the path between the FIQR total score and perceived stress. This result was not found to be significant for the other sub-indices (see Figure 1a). A similar model was tested for MDD (R^2^ = 0.76, F [7,85] = 16.66, *p* < 0.001) showed a marginally significant effect for the non-acceptance index (β = 0.18, *p* = 0.06) and for the FIQR total score (β = 0.19, *p* < 0.001). Estimates of the indirect effect of non-acceptance (β = 0.03) had a CI (0.00–0.06) that was significantly greater than zero, indicating that non-acceptance partially mediated the path between FIQR total score and PHQ-9 total score; the other sub-indices were not found to be mediators (see Figure 1b).

## 4. Discussion

In this study, we examined the role of emotion regulation in the comorbidity between fibromyalgia and psychological distress (depression and perceived stress) among a treatment-seeking sample. We found significant associations between measures of fibromyalgia, psychological distress, and emotion regulation difficulties. Importantly, difficulties in emotion regulation partially mediated the association between fibromyalgia symptoms and perceived stress.

Studies indicate very high comorbidity between fibromyalgia and psychological distress [27], the causes of which are somewhat unclear. To date, various hypotheses have been suggested, i.e., that fibromyalgia causes emotional distress or vice versa, as well as the suggestion that psychological symptoms are an integral component of one broad mind-body disorder [28]. Our findings indicate that emotion regulation may be difficult for those with fibromyalgia symptoms. Importantly, non-acceptance of emotional responses seems to play a particularly important role in fibromyalgia and should be examined in light of the stigmatization experience of patients [29]. Individuals with fibromyalgia often face doubts and suspicion on the part of the medical establishment as well as others in their environment who may question the validity of their diagnosis. Our findings are in line with studies showing the importance of emotions for MUS patients [12,13]. Our findings are also in line with the “Match-Mismatch Model”, which argues that specific emotion regulation strategies may be more/less beneficial depending on one’s emotional processing style. This model was also examined among fibromyalgia patients [17]. Notably, the emotion regulation scale used in this study (DERS [24]) is in line with the third wave of cognitive-behavioral therapy (e.g., mindfulness, ACT). The scale emphasizes concepts such as acceptance, non-judgment, and non-reactivity, all of which seem to be highly relevant for such a chronic and debilitating condition as fibromyalgia [8].

Importantly, in this study, emotion regulation mediated the association between fibromyalgia symptoms and perceived stress. When the sub-indices that make up the emotional regulation difficulties index were examined, the non-acceptance of emotional responses index was found to be the only one that mediates the association between fibromyalgia and perceived stress and fibromyalgia and depression (it should be noted that this was found to be marginally significant). A vast body of literature shows that emotion regulation is considered a powerful, transdiagnostic mechanism in psychopathology [30]. Here, we show that the association between fibromyalgia symptoms and psychological distress is partially explained by emotion dysregulation and especially by non-acceptance of emotion responses. A possible explanation for this mediation process may be that fibromyalgia symptoms are often quite difficult to tolerate and cope with. Fibromyalgia patients may feel frustrated and even helpless in light of the pain and fatigue they experience. Those who indeed have difficulty regulating these emotions may subsequently experience psychological distress in the form of a negative mood, stress, or decreased quality of life.

Our lack of findings regarding the role of other sub-indicates of emotion regulation as mediators’ components requires examination in further studies and especially in RCTs that examine emotion regulation as a mechanism of change in treatment for fibromyalgia patients. In particular, our lack of findings regarding lack of emotional awareness may be explained by the idea that fibromyalgia patients know their distress quite well but lack sufficient skills to adaptively cope with it. In other words, awareness may be a necessary but insufficient condition for alleviating mental distress in fibromyalgia.

Limitations of the study include the use of self-report questionnaires and a reliance on a treatment-seeking sample, which may limit generalizability. In addition, our study did not include a group of healthy controls or a control group of patients diagnosed with other diseases or mental illnesses. Thus, we cannot establish whether the processes identified here are specific to fibromyalgia patients. Therefore, we recommend that future studies include a control or comparison group. Moreover, in our study, participants were required to obtain/show an existing diagnosis from a licensed rheumatologist. Adding a professional who could examine participants as part of the study would have potentially improved the quality and validity of the diagnosis. We recommend that future studies employ a clinical assessment of participants.

Despite these limitations, this study has clinical and theoretical significance. It is one of only a few studies to date attempting to examine a mechanistic model of emotion regulation as an explanation for psychiatric distress in fibromyalgia. Emotion regulation may serve as an important therapeutic target in fibromyalgia, for example, in third-wave CBT interventions (e.g., MBSR), which have been shown to be effective in chronic pain disorders [31]. Our focus on the role of specific emotion regulation strategies may further inform therapists and improve care.

## Figures and Tables

**Table 1:** Associations between study variables.

	Major Depression (PHQ-9)	Perceived Stress (PSS)	FM (FIQR)	Difficulties in Emotion Regulation (DERS)	Nonacceptance of Emotional Responses (DERS)	Difficulty Engaging in Goal-Directed Behavior (DERS)	Impulse Control Difficulties (DERS)	Lack of Emotional Awareness (DERS)	Limited Access to ER Strategies (DERS)	Lack of Emotional Clarity (DERS)
Major depression (PHQ-9)		0.752 ***	0.719 ***	0.373 ***	0.507 ***	0.287 **	0.367 ***	−0.139	0.325 ***	0.280 **
Perceived stress (PSS)			0.698 ***	0.480 ***	0.618 ***	0.316 **	0.516 ***	−0.172	0.452 ***	0.246 *
FM impact (FIQR)				0.351 ***	0.436 ***	0.279 **	0.322 **	−0.035	0.230 *	0.353 **
Difficulties in emotion regulation (DERS)					0.798 ***	0.691 ***	0.804 ***	0.406 ***	0.851 ***	0.490 ***
Nonacceptance of emotional responses (DERS)						0.527 ***	0.684 ***	0.005	0.721 ***	0.333 **
Difficulty engaging in goal-directed behavior (DERS)							0.492 ***	0.070	0.703 ***	0.182
Impulse control difficulties (DERS)								0.021	0.762 ***	0.417 ***
Lack of emotional awareness (DERS)									0.065	0.232 *
Limited access to ER strategies (DERS)										0.246 *
Lack of emotional clarity (DERS)										

*** *p* < 0.001 ** *p* < 0.01 * *p* < 0.05.

## Data Availability

The data presented in this study are available upon request from the corresponding authors. The data are not publicly available due to privacy.
